# Varicocele-Mediated Male Infertility: From the Perspective of Testicular Immunity and Inflammation

**DOI:** 10.3389/fimmu.2021.729539

**Published:** 2021-08-31

**Authors:** Yiwei Fang, Yufang Su, Jia Xu, Zhiyong Hu, Kai Zhao, Chunyan Liu, Huiping Zhang

**Affiliations:** Institute of Reproductive Health, Tongji Medical College, Huazhong University of Science and Technology, Wuhan, China

**Keywords:** varicocele, male infertility, sperm, testicular immunity, inflammation

## Abstract

**Background:**

Varicocele (VC) is present in 35 - 40% of men with infertility. However, current surgical and antioxidant treatments are not completely effective. In addition to oxidative stress, it is likely that other factors such as testicular immune microenvironment disorder contribute to irreversible testicular. Evidence suggests that VC is associated with anti-sperm antibodies (ASAs), spermatogenesis and testosterone secretion abnormalities, and testicular cytokine production. Moreover, inhibition of inflammation can alleviate VC-mediated pathogenesis. The normal function of the testis depends on its immune tolerance mechanism. Testicular immune regulation is complex, and many infectious or non-infectious diseases may damage this precision system.

**Results:**

The testicular immune microenvironment is composed of common immune cells and other cells involved in testicular immunity. The former includes testicular macrophages, T cells, dendritic cells (DCs), and mast cells, whereas the latter include Leydig cells and Sertoli cells (SCs). In animal models and in patients with VC, most studies have revealed an abnormal increase in the levels of ASAs and pro-inflammatory cytokines such as interleukin (IL)-1 and tumor necrosis factor (TNF)-alpha in the seminal plasma, testicular tissue, and even peripheral blood. It is also involved in the activation of potential inflammatory pathways, such as the nucleotide-binding oligomerization domain-like receptor family pyrin domain containing (NLRP)-3 pathway. Finally, the development of VC-mediated infertility (VMI) may be facilitated by abnormal permeability of proteins, such as claudin-11, that constitute the blood-testis barrier (BTB).

**Conclusions:**

The testicular immune response, including the production of ASAs and inflammatory factors, activation of inflammatory pathways, and destruction of the BTB may be involved in the pathogenesis of VMI it is necessary to further explore how patient outcomes can be improved through immunotherapy.

## Introduction

Approximately 15% of couples worldwide have infertility; half of these cases are attributable to male factors. Varicocele (VC), a vascular disease characterized by abnormal enlargement of the pampiniform plexus veins, is a highly treatable cause of infertility observed in 35–40% of men with infertility (1). VC is left-sided in at least 85% of cases (2); right-sided VCs are rare (3). Surgical ligation or embolization of the spermatic cord vein can improve semen quality, sperm DNA integrity, mitochondrial activity, and assisted reproductive cycle outcomes (4). It has been suggested that VC-mediated infertility (VMI) is not caused by a single factor but is the result of the synergy of genetic and other molecular factors, such as hypoxia, oxidative stress, and nutrient deprivation (5). However, some patients fail to regain fertility after surgery, antioxidant therapy, or other treatments. Interestingly, Mazdak Razi et al. (6) proposed that VMI is caused by endoplasmic reticulum stress and the unfolded protein response, which promote testicular cell apoptosis, and could be treated with antioxidants and anti-inflammatory molecules. In addition to the above factors, there are likely other factors that contribute to irreversible testicular damage. Evidence suggests that VC is associated with anti-sperm antibodies (ASAs) (7), spermatogenesis and testosterone secretion abnormalities (8), and cytokine production (9) in the testes. Moreover, inhibition of inflammation can alleviate VC-mediated pathogenesis (10). The normal function of the testis depends on its immune tolerance mechanism (11). The complex yet precise system of testicular immune regulation may be compromised by various infectious or non-infectious diseases.

The exact mechanism by which VC causes infertility remains unknown. It is generally accepted that the pathogenesis of VMI is complex and multifactorial (12, 13). Currently, immunotherapy is rarely applied to VMI. This review explores the mechanisms of VMI from the perspective of reproductive immunology. In the first half of this article, we review the composition of the testicular immune microenvironment under normal physiological conditions, and in the second half, we focus on the potential immunological pathogenesis of VMI, including the role of ASAs, the blood–testis barrier (BTB), cytokines, and inflammatory pathways. Rather than denying the important role of oxidative stress, this review explores the reproductive immune mechanism of VMI from an alternative perspective and provides ideas for future research and potential treatment of VMI.

## Testicular Immunity

Mammalian testes create a unique immune environment; that is crucial for testicular function. The testis is a significant immune-sparing site that protects immunogenic germ cells from adverse effects of the immune response (14). Both local immunosuppression and systemic immune responses may be involved in maintaining testicular immunity (15). Pattern recognition receptors (PRRs) play a significant role in the testicular innate immune response. The BTB prevents harmful substances from entering the seminiferous epithelium and prevents sperm antigens from escaping into the seminiferous tubules and causing autoimmune reactions. Moreover, testicular cells secrete immunosuppressive factors and negative regulatory factors considered to be associated with several diverse immune cells (16), including testicular macrophages, lymphocytes, dendritic cells (DCs), and mast cells.

## Common Immune Cells

### Testicular Macrophages

Macrophages are tissue-specific immune cells that are widely present in various tissues; their functions differ depending on the site. Testicular macrophages are the major immune cells in the testes, accounting for approximately 20% of murine testicular stromal cells (17), which regulate the development of rat Leydig cells and steroidogenesis (18, 19). Specifically, testicular macrophages play a key role in fetal testicular organogenesis (20), testosterone synthesis (21), spermatogenesis (22), and testicular immunosuppression (23). Notably, the inflammatory response of testicular macrophages is lower than that of blood macrophages (24–26). Therefore, testicular macrophages are regarded as testicular guardians (27). In other words, testicular macrophages are characterized by a higher degree of immunosuppressive activity than macrophages from other tissues. Testicular macrophages are mainly polarized into pro-inflammatory (M1) and inhibitory-inflammatory (M2), the latter of which is characterized by high levels of anti-inflammatory cytokines, such as interleukin (IL)-10 and transforming growth factor-beta (TGF-β) (28). Furthermore, testicular macrophages in rats are less responsive to pathogen stimulation; and constitutively produce anti-inflammatory cytokines (29, 30), such as IL-10, which are essential for the prevention of organ-specific autoimmune inflammation (8). In contrast, under the influence of some negative regulatory factors, macrophages polarize to M1 and secrete high levels of pro-inflammatory cytokines, such as IL-1β and TNF-α (31).

### Testicular Lymphocytes

Lymphocytes are cells with immune recognition functions. They can be classified as T cells, B cells, and natural killer (NK) cells according to their migration, surface molecules, and function. T cells are vital regulators of cellular and humoral immunity and can be distinguished as CD4^+^ T cells and CD8^+^ T cells based on their surface receptors. T cell receptors recognize antigenic proteins released by pathogens. Under normal physiological conditions, CD8^+^ T cells are the predominant lymphocytes in the testicular interstitial space (32). Regulatory T cells (T-regs) promote peripheral immune tolerance and exhibit immunosuppressive properties in the vasectomy model (33). Murine models of allogeneic islet cell transplantation have also exhibited the immunosuppressive effects of testicular T-regs characterized by CD4^+^ CD25^+^ (34, 35). T-regs inhibit the activation of effector T cells under normal physiological conditions (35) and increase T cells counts under inflammatory conditions (36). These observations suggest that T-regs contribute to testicular immunity. Induction of T cell apoptosis is one of the methods by which the testes suppress the immune response, and the Fas/Fas ligand (Fas/FasL) system is highly expressed in the testes (37, 38). Programmed deathligand-1(PDL-1) is significantly expressed in male germ cells. The Fas/FasL and the programmed death-1 (PD-1)/PDL-1 system can induce T cell apoptosis, thereby and protecting against islet allogeneic immune damage (39, 40). Testicular NK cells also have immunoregulatory properties, but there have been only a few studies on these cells. Previous studies have used methods such as flow cytometry to confirm that there are no B cells in the testes under physiologically normal conditions (41).

### DCs and Mast Cells

DCs are the most functional, professional antigen-presenting cells in the body and are highly efficient at ingesting, processing, and presenting antigens. DCs occur in the interstitial spaces of the testes and are immature under normal physiological conditions. The number of DCs increases significantly and they express maturation markers in the inflammatory state (42, 43), indicating that DCs play an important role in testicular autoimmunity (44). DCs minimize autoimmune responses by tolerating T cell auto-antigens under normal physiological conditions (45). However, the mechanism underlying their effects in the testes requires further study. According to Banchereau’s study (46), the maturation of testicular immunosuppressive T cells requires the assistance of mast cells.

Mast cells secrete a variety of cytokines and are involved in immune regulation and immediate hypersensitivity (type I allergy). The number of mast cells in the testes is small under normal physiological conditions. However, mast cells are heavily differentiated during inflammatory activation (47). Abnormal spermatogenesis is associated with an increased number of mast cells (48). A randomized controlled trial has demonstrated that Zaditen, a mast cell blocker, improves semen parameters, chromatin integrity, and pregnancy rates after VC surgery (49). Research on mast cells is limited, and the function of mast cells in testicular immunity is not completely understood. However, this review demonstrates, their important role in testicular immune disorders and their effects on sperm quality parameters.

## Other Cells Involved in Testicular Immunity

### Leydig Cells

In addition to regulating male sex differentiation and fertility by producing testosterone, Leydig cells can also indirectly contribute to the testicular immune microenvironment. Representing the majority of mesenchymal cells, Leydig cells have congenital antiviral infection function in rats (50, 51). Furthermore, testosterone inhibits systemic immune responses to autoantigens (52, 53). Androgen receptor-deficient murine Sertoli cells (SCs) impair immune exemption in the seminiferous tubules, suggesting that testosterone also plays a local role in maintaining testicular immune privilege (54). Leydig cells also play an important role in the innate immune defense of the testes. In microbial invasion, Leydig cells synthesize and release antiviral factors, such as TNF-α and interferon (IFN)- α, or anti-inflammatory factors, such as IL-6 and IL-1β (55, 56).

#### SCs

As part of the BTB, SCs are crucial for testicular immune privilege. Tight junctions form between adjacent SCs in the testicle; this is the basis of the BTB, blocking contact between systemic immune cells and spermatogenic cells in the stroma, thereby producing immune tolerance (57, 58). Once the tight junctions are destroyed, the BTB is weakened. More seriously, as demonstrated in mice, sperm cell differentiation is altered with significant germ cells loss, suggesting that the BTB/SC barrier effectively prevents humoral immune responses to late germ cells in normal testes (56).

Various cytokines secreted by SCs also form a part of the testicular immune microenvironment. TGF-β is mainly produced by testicular SCs in testis for immune suppression (59). SCs also express large amounts of activin A and B (60). The former is similar in structure to TGF-β, which inhibits the expression of pro-inflammatory cytokines (including IL-1 and IL-6), thereby inhibiting testicular inflammation. Once Toll-like receptors (TLRs) expressed on SCs are activated, a large number of pro-inflammatory factors are released (39). Gas6 is a functional ligand of the Tyro 3 receptor tyrosine kinase (RTK) subfamily, and its tyrosine kinase receptors TAM are constitutively expressed in murine testes (61). It mediates the ability of SCs to phagocytose apoptotic germ cells and plays a key role in testicular immunity (40, 62).

## The Immune Mechanism of VC

The current consensus is that VMI is mainly caused by hypoxia and oxidative stress, as clarified in a review by Jensen (12), but some phenomena cannot be fully explained by these factors alone. Numerous studies have suggested that inflammation and immunity play a role as mediators, and any abnormal changes in the immune links of the testes may have a negative impact. Testicular macrophages are involved in the pathogenesis of various inflammatory diseases. Experimental autoimmune orchitis models have shown substantial macrophage infiltration and secretion of numerous inflammatory factors (25, 63). Some researchers have found that the pathological changes in the testes of experimental autoimmune orchitis models are similar to those in VC, both are associated with abnormalities of permeability and structure of the BTB structure (64). Our previous study showed that the inflammasome, NLRP3, is activated in testicular macrophages, producing a large amount of IL-1β and inhibiting the normal synthesis of testosterone (65).

### ASAs

ASAs are present in 1-2% of fertile men and 5-15% of men with infertility (66). Sperm immunoglobulin (Ig) levels in the seminal plasma of men with infertility and VC were higher than those in men with infertility but without VC (67, 68). The positive rate of mixed antiglobulin reaction (MAR)-IgG in the seminal plasma of patients with VMI was more than three times that of normal seminal plasma (66). In patients with improved semen quality after surgery, the level of seminal ASAs decreased accordingly (69, 70). Animal experiments also showed similar results; i.e. the ASA of the VC rats model increased (71). The incidence of anti-sperm immune responses is associated with increased chromosomal abnormalities in the gametes. There was a significant positive correlation between the MAR-IgG percentage and sperm DNA fragmentation rate. ASAs can agglutinate sperm and bind to apoptosis-related proteins (e.g., caspase 3 and hsp70), leading to apoptosis in sperm cells (72).

**Figure 1:**
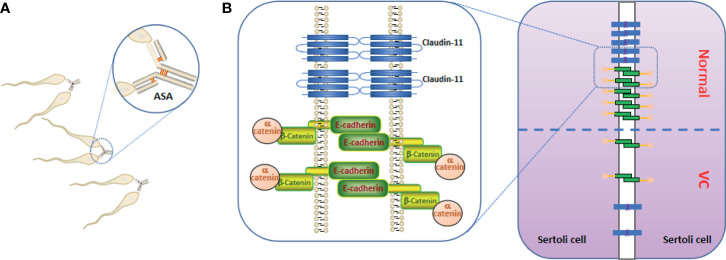
Anti-sperm antibodies (ASAs) and blood testes barrier (BTB). **(A)** ASAs bind to sperm, resulting in sperm agglutination and sperm motility reducing. **(B)** the important constituent proteins (claudin-11, E-cadherin, and α-catenin) that constitute the BTB are reduced in the testes of Varicocele (VC), resulting in increased BTB permeability.

ASAs affect male fertility through different mechanisms, and largely correlates with sperm autoimmunity and ASA antigen specificity (**Figure 1A**). The main role of the testicular autoimmune response is to reduce sperm movement and agglutination (73). An anti-spermatic immune response is associated with decreased sperm function and sperm quality parameters, such as DNA breaks. One study found that sperm quality in patients with ASA (+) VC is significantly reduced and correlates with the VC grade (66). Simultaneously, sperm damage is more severe in patients with ASA positive VC. The spermatozoa count in grade 2 ASA-positive VCs patients was less than half of that in ASA negative patients, whereas the reactive oxygen species (ROS) level in ASA (+)patients with infertility was 2.8 and 3.5 times higher than that of ASA (-) patients, respectively (5). However, the results of ASA testing in patients with infertility were similar regardless of whether the patient had been diagnosed with VC (2). In other words, VC may be an important cofactor for ASA production. VC increases the likelihood of immune infertility; after testicular trauma, the probability of immune infertility in patients with VC increases by a factor of 2 (66). Therefore, VC may not be the direct cause of ASA production, but VC is a synergistic factor that leads to immune sterility. However, Bozhemov et al. (66) suggested that VC is not a direct cause of the sperm autoimmune response; but a cofactor that increases the risk of ASA production. In VC patients, the odds ratio for immune infertility tripled after testicular trauma.

## Permeability of the BTB

In the VC animal model, Pan et al. (74) and Raĭtsina et al. (64) reported a significant disruption of the BTB, and the latter also found lymphocytes sensitized to spermatozoa antigens in the lymphatic organs of experimental rats. Soares et al. (75) suggested that the receptor for activated C kinase 1 up-regulation, induced by VC, may also be involved in the phosphorylation of focal adhesion kinase, thus affecting the dynamics of the BTB and apical ectoplasmic specialization. Immune factors in VC act on the adherent tight junction molecule of the BTB, increasing the permeability of the BTB. The immune factor subsequently enters the seminiferous tubule to destroy spermatogenic cells (73, 76–79). Simultaneously, sperm antigens may be exposed to the circulating blood. Claudin-11, a tight junction protein expressed by SCs, E-cadherin, and α-catenin have been shown to participate in cell adhesion, and all molecules are involved in the formation of the BTB but the levels in the seminiferous tubules were significantly down-regulated (**Figure 1B**) (8, 74, 80, 81).

## Inflammatory Factors and Pathways

### IL

Multiple studies have demonstrated the central role of IL-1α and IL-1β in a number of autoinflammatory diseases (82–85). In the testes, IL-1 regulates spermatogenesis through autocrine and paracrine expression, and overexpression of IL-1 is critical for improving VMI. Studies have shown that IL-1 expression in the testes has increased in VC animal models (86, 87). Kim’s study (88) and Sahin et al. (89) found that the expression of both IL-1α and IL-β increased in the testes of the VC model, which could be reversed by treatment with herbs. However, case-control studies found that, compared with that in the control group, the level of IL-1β in the seminal plasma of patients with VC did not change significantly (9, 55, 90). (**Table 1**) This contrasts with the increased IL-1 levels typically observed in the testicular tissues of patients with VMI. A potential explanation for this is that the liquid components of the semen originate from multiple sites, including the seminal vesicle gland and prostate, and may dilute the levels of the original substances in the testicles. Therefore, IL-1 is most likely a factor that causes infertility in patients with VC.

**Table 1:** Summary of cytokine reported in VC of human and animal model.

Cytokine	Species	Group	Sample type	Regulation	References
IL-1	Rats	VC & Sham	Testicular tissue	Up	(86, 87)
IL-1α	Rats	VC & Sham	Testicular tissue	Up	(8, 89)
IL-1β	Rats	VC & Sham	Testicular tissue	Up	(88, 89)
IL-1β	Homo sapiens	Infertility with VC & Normal	seminal plasma	NSS	(55, 90)
IL-1β	Homo sapiens	VC & without VC	seminal plasma	NSS	(9)
IL-6	Rats	VC & Sham	Testicular tissue	Up	(8, 88, 91, 92)
IL-6	Rats	VC & Sham	Serum	Up	(91)
IL-6	Homo sapiens	Infertility with VC & Normal	seminal plasma	Up	(55, 93)
IL-6	Homo sapiens	VC & Fertility	seminal plasma	Up	(94)
IL-6	Homo sapiens	VC & without VC	seminal plasma	Down	(9)
IL-8	Homo sapiens	VC & without VC	seminal plasma	NSS	(9)
IL-8	Homo sapiens	VC & without VC	seminal plasma	Up	(55, 95)
IL-10	Homo sapiens	VC & without VC	seminal plasma	Up	(9)
IL-17A	Homo sapiens	VC & without VC	seminal plasma	NSS	(9)
IL-18	Homo sapiens	Infertility with VC & Normal	seminal plasma	NSS	(96)
IL-18	Homo sapiens	Infertility with VC & Normal	seminal plasma	Up	(97)
IL-37	Homo sapiens	Infertility with VC & Normal	seminal plasma	Up	(97)
TNF-α	Rats	VC & Sham	Testicular tissue	Up	(8, 71, 88, 92)
TNF-α	Homo sapiens	Infertility with VC & Normal	seminal plasma	NSS	(90)
TNF-α	Homo sapiens	Infertility with VC & Normal	seminal plasma	Up	(55, 94)
TNF-α	Homo sapiens	VC & without VC	seminal plasma	NSS	(9)
TNF-α	Homo sapiens	VC & Normal	seminal plasma	NSS	(98)
IFN-γ	Rats	VC & Sham	Testicular tissue	Up	(91, 92)
IFN-γ	Rats	VC & Sham	serum	Up	(91)
IFN-γ	Homo sapiens	VC & without VC	seminal plasma	Down	(9)
IFN-γ	Homo sapiens	Infertility with VC & Normal	seminal plasma	NSS	(96)
TGF-β	Rats	VC & Sham	Testicular tissue	Up	(71)

Up, Up-regulation of cytokine levels; Down, Down-regulation of cytokine levels; NSS, Not statistically significant; VC, varicocele.

IL-6 is produced rapidly and briefly in response to infection and tissue damage, but the persistent dysregulation of IL-6 synthesis plays a pathological role in chronic inflammation and autoimmunity (99). Most studies (8, 55, 88, 91–94) support the view that the level of IL-6 is increased in VC animal models or patients with VMI (**Table 1**). The concentration of IL-6 in the seminal plasma of patients with VC is much higher than that in fertile men (55, 93, 94), but lower in Finelli’s study (9). However, the study conducted by Finelli et al. included only patients with VC, excluding those with VMI the focus of our review. This may be one of the main reasons why the levels of IL-1β, IL-6, IL-8, TNF-α, and IFN-γ found in that study are inconsistent with those found in other studies. In patients with VMI, it has been reported that levels of IL-6 and ROS are elevated and that total oxidative binding energy is decreased. *In vitro*, IL-6 reduces sperm motility, possibly attributed to excessive production of nitric oxide (NO) (100). IL-1 and IL-6 have inhibitory effects on the acrosomal reaction similar to those of TNFα (93). In short, IL-6, like IL-1, may be an important immunoregulatory factor affecting fertility in patients with VC.

The number of white blood cells in seminal plasma is significantly correlated with IL-8, also known as C-X-C motif chemokine ligand (CXCL) -8 (55, 73, 101). Compared with those in the control group, the levels of IL-8 in the seminal plasma of patients with VC was found to be higher (55, 95). The mechanism related to IL-6 and IL-8 has not yet been reported. Similarly, IL-17A and IL-18, as well as anti-inflammatory factors IL-10 and IL-37, have also been reported in a series of studies (9, 96–98) (**Table 1**). In general, the current research on IL-8, IL-10, IL-17A, IL-18 and IL-37 is limited, and it is difficult to draw definite conclusions on the role of ILs in VC. Further investigation on this topic is required, is needed in the future, particularly through high-quality human studies.

### TNF-α, IFN-γ, and TGF-β

Multiple studies have reported an increase in TNF-α levels in both patients with VC and in animal models (**Table 1**). For other two studies (9, 98) did not find significant changes in TNF-α levels because they included patients different from those in the other studies, as described above. TNF-α alters mitochondrial function, increases NO production, and is inversely related to sperm motility. It also increases the production of malondialdehyde and inhibits spontaneous and induced acrosomal reactions (73). The role of pro-apoptotic TNF-α in the pathogenesis of VC-mediated dysfunction is currently under intense study (93). Animal studies demonstrated that IFN-α levels increased in the testes or serum, but human studies did not support this finding, possible reasons have been explained in IL-1. (**Table 1**) Habibi et al. (91) found that IFN-γ levels in serum and testicular tissue were increased in rat VC models, suggesting that VC has a detrimental, time-dependent effect on cytokine levels and decreases the number of SCs, and spermatogonia, as well as the seminiferous tubules diameter and sperm indices. TGF-β balances pro-inflammatory and anti-inflammatory effects by reducing cell growth of immune precursors and plays a key role in immune tolerance (102). Similar to those of most cytokines, TGF-β levels were also reported to be increased in the testes of VC model (**Table 1**) (71). Together, these results suggest that TNF-α and IFN-γ are potential markers of testicular damage in patients with VC.

Overall, most of the available data from patients with VC or animal VC models support the observation of elevated levels of pro-inflammatory factors in the testicular tissues and seminal plasma, to varying degrees. In particular, IL-1, IL-6, TNF-α, and IFN-γ may be key immunoregulatory factors for testicular injury in patients with VC. Simultaneously, some anti-inflammatory factors, such as IL-10, IL-37, and TGF-β, tend to be upregulated; this may be the result of the normal feedback mechanism of the body. These anti-inflammatory factors may also be of consequence to future treatment strategies, but their authenticity must first be confirmed by further studies.

### Inflammatory Pathways

PRRs can be divided into five categories, including TLRs, located on the cell membrane, and NLRs, located in the cytoplasm (103). They occur mainly on immunocytes such as macrophages, DCs, and rarely non-immunized cells. Thus, once PRRs are activated by pathogen-associated molecular patterns (PAMPs) or danger-associated molecular patterns (DAMPs), the signal will eventually be transmitted to the downstream target gene, which regulates immunity and inflammation (104). PRRs and their specific ligands are listed in detail in a review by Wang et al. (103).

TLR2, one of the TLRs, can also recognize PAMPs and/or DAMPs (105), the latter including heat shock proteins (HSP60, HSP70, Gp96), advanced glycation end-products, high mobility group box 1, and serum amyloid A (103, 106). The average relative expression of TLR2 in the semen of men with infertility and VC was twice as high as that in fertile men, but the difference was not statistically significant (107).

NLRP3 is a subtype of the NLR family, that can be directly triggered by cytoplasmic DAMPS, such as peptides, DNA, and RNA (103). NLRP3 inflammasomes are composed of cytoplasmic sensor molecules, such as pyrin domain-containing protein 3, adaptor proteins (*e.g.*, caspase-recruiting domain, ASC, and apoptosis-associated speck-like proteins), and effector proteins (*e.g.*, pro-caspase-1). NLRP3 and ASC promote the cleavage of pro-caspase-1 and form an active complex, which triggers the cleavage of pro-IL-1β into mature IL-1β (65). The VC model showed a significant increase in NLRP3 gene expression after partial ligation of the left renal vein (108). Interestingly, this trend was reversed by the administration of Resveratrol (3,5,4′-trihydoxy-trans-stilbene), which is an anti-inflammatory, anti-apoptotic compound in some plants (109). We previously found a significant up-regulation of prokineticin 2 (PK2) in the VC animal model (110), whereas in the orchitis model PK2 promoted IL-1β secretion *via* the NLRP3 pathway (111). We speculate that this process is also involved in the onset of VMI, although this hypothesis has not been validated with rigorous experiments.

## Future Perspective

The current treatment approach of VC is mainly surgical, but a certain proportion of patients find it difficult to regain natural fertility even after surgery. Whether normal fertility can be restored in these patients through immunotherapy is worth further exploration. Although the studies reviewed here have reported various abnormalities of immune-related cells or active molecules in the testicular tissue or seminal plasma of patients with VC, little is known about how these factors interact or function. However, there are currently at least three strategies to protect the testicular immune microenvironment in cases of VMI reduction of the production of ASAs and removal of ASAs from the body, reduction of BTB permeability, i.e., reduction of sperm antigen exposure; maintenance of the balance of pro-inflammatory and anti-inflammatory molecules and cells in testes, as well as inhibition of related inflammatory pathways. It should be emphasized that the molecular targets of these three strategies are not clearly defined and need further exploration.

## Conclusions

The mechanism by which VC induces male infertility is complex and multi-factorial. However, at its core, it is a disorder of the testicular immune microenvironment caused by various injury factors. Where testicular immunity and inflammation are concerned, it mainly involves the production of ASAs, an increased permeability of the BTB caused by abnormal proteins, the release of a series of inflammatory factors, and the activation of inflammatory pathways. Although some protective molecules, such as anti-inflammatory cytokines, are up-regulated, this is not enough to combat the negative regulatory of damaging factors. Given that immune mechanisms are involved in the pathogenesis of VMI, it is necessary to investigate the use of immunotherapy to improve patient outcomes in the future.

## Author Contributions

YF, YS, and HZ designed the work. YF, YS, JX, and ZH collected and analyzed the relevant reports. YF, YS, and JX wrote the paper. KZ and CL provided substantial contributions to improve the content of the article. All authors contributed to the article and approved the submitted version.

## Funding

This work was funded by National Key R&D Program of China (Grant numbers: 2018YFC1004300, 2018YFC1004304), National Natural Foundation of China (Grant numbers: 81701539).

## Conflict of Interest

The authors declare that the research was conducted in the absence of any commercial or financial relationships that could be construed as a potential conflict of interest.

## Publisher’s Note

All claims expressed in this article are solely those of the authors and do not necessarily represent those of their affiliated organizations, or those of the publisher, the editors and the reviewers. Any product that may be evaluated in this article, or claim that may be made by its manufacturer, is not guaranteed or endorsed by the publisher.
